# A Three-Dimensional Deformation Monitoring Method: Combining Optical Deformation Monitoring Based on Regression Models and GB-SAR Interferometry

**DOI:** 10.3390/s24061754

**Published:** 2024-03-08

**Authors:** Yanbo Cheng, Yuanhui Mo, Haifeng Huang, Tao Lai

**Affiliations:** School of Electronics and Communication Engineering, Shenzhen Campus, Sun Yat-sen University, Shenzhen 518107, China

**Keywords:** three-dimensional deformation, ground-based synthetic aperture radar, fusion data, regression model

## Abstract

This paper introduces a method for quantifying the three-dimensional deformation of ground targets and outlines the associated process. Initially, ground-based synthetic aperture radar was employed to monitor the radial deformation of targets, and optical equipment monitored pixel-level deformation in the vertical plane of the line of sight. Subsequently, a regression model was established to transform pixel-level deformation into two-dimensional deformation based on a fundamental length unit, and the radar deformation monitoring data were merged with the optical deformation monitoring data. Finally, the fused data underwent deformation, resulting in a comprehensive three-dimensional deformation profile of the target. Through physical data acquisition experiments, the comprehensive three-dimensional deformation of targets was obtained and compared with the actual deformations. The experimental results show that the method has a relative error of less than 10%, and monitoring accuracy is achieved at the millimeter level.

## 1. Introduction

In recent years, frequent geological disasters have arisen from the disruption of geological structures triggered by crustal movements such as volcanoes and earthquakes, shifts in climate patterns due to global warming, and human activities such as mineral extraction and deforestation [1,2]. Beyond natural calamities, deformation issues affect structures such as buildings. Construction facilities such as bridges, dams, and high-rise buildings commonly undergo specific deformations and settlements throughout their life cycles, sometimes leading to severe incidents, e.g., collapse [3].

Geological disasters typically unfold gradually, with deformation accumulating over the long term rather than occurring suddenly [4]. Preceding major disaster events, potential hazards often undergo gradual and incremental deformations, accumulating slowly until instability arises [5,6]. Identifying these subtle deformation processes in a timely manner and issuing early warnings can enable targeted disaster mitigation, effectively preventing casualties and economic losses [7]. Therefore, it is necessary to carry out the long-term, continuous, and real-time monitoring of slopes and mines, dilapidated buildings, and large buildings with landslide and collapse risks, evaluate their stability, and make early warnings if necessary [8].

Differential interferometric synthetic aperture radar (DInSAR) has been a key player in surface monitoring, boasting high precision, extensive coverage, and all-weather capabilities since its inception. However, challenges persist for space-based SAR in terms of measurement accuracy and resolution, attributed to issues such as time decorrelation, long spatial baselines, and constraints on electromagnetic wave irradiation angles [9,10]. Ground-based synthetic aperture radar (GB-SAR), following in the footsteps of space-based and airborne SAR, has gained widespread adoption for high-precision deformation monitoring in areas affected by natural disasters, dams, alpine glaciers, and pier structures. This popularity is attributed to its advantages, including a short space-time baseline, high resolution, a brief measurement period, and ease of operation [11,12,13].

As an emerging technology, GB-SAR provides detailed and accurate measurements of ground deformation [14,15]. Despite the significant advancements in GB-SAR research in recent years, challenges persist [16]. Notably, GB-SAR’s limitation in capturing only the projection component of the target’s deformation variable along the radar radial direction can result in underestimated deformations. This limitation becomes particularly evident when a substantial angle exists between the target’s deformation direction and the radar radial direction, causing the projection of even significant deformations to appear very small in GB-SAR [17,18]. This discrepancy poses the risk of missed alarms and potential hazards [19]. In order to address this issue with GB-SAR, some scholars have attempted multi-station monitoring to capture the multi-dimensional deformation of a target. However, this approach introduces increased equipment costs and deployment challenges. Moreover, the current accuracy of this method still remains at the centimeter level [20].

In addition to GB-SAR deformation monitoring, optical imaging deformation technology finds widespread applications in various fields, including materials science and medicine [21]. Optical deformation monitoring captures relevant information in the vertical plane of line of sight (LOS). We aim to leverage optical images to complement the third-dimensional information not attainable through radar monitoring. This approach seeks to achieve comprehensive three-dimensional deformation analysis for ground targets, minimizing the likelihood of missed alarms during monitoring. Furthermore, it provides essential technical support for natural disaster warnings and building deformation monitoring [22,23].

In this paper, a technique for obtaining the comprehensive three-dimensional deformation of ground targets is proposed. Firstly, based on GB-SAR and optical images, radar deformation monitoring, and optical deformation monitoring were carried out, respectively, and a nonlinear fitting regression model was established. The pixel-level deformation obtained by optical deformation monitoring was converted into deformation under the basic length unit, and the deformation monitoring data of the two were fused to solve the comprehensive three-dimensional deformation of targets finally.

Key innovations in this paper include the following:

1. Proposing a method to convert pixel deformation quantity from optical deformation monitoring into deformation quantity under a basic length unit (mm).
2. Conducting three-dimensional deformation inversion by integrating optical and radar deformation information.
3. Establishing a three-dimensional solution model and validating the feasibility and superiority of three-dimensional deformation inversion through measured data. In single-station monitoring scenarios, the accuracy of deformation monitoring within tens of meters reaches the millimeter level, with a controlled relative error below 10%.

The paper is organized as follows: Section 2 delves into the principles of deformation monitoring using GB-SAR interferometry measurements. In Section 3, we present a comprehensive overview of the methods proposed in this article. This includes a novel optical deformation monitoring technology based on fitting a regression model, along with a three-dimensional deformation inversion technology that integrates optical and radar images. Section 4 validates the feasibility and effectiveness of the proposed method through experimental testing. In conclusion, Section 5 provides a comprehensive summary and the concluding remarks of the paper.

## 2. GB-SAR Deformation Monitoring

The image obtained by GB-SAR monitoring is a single-look-complex (SLC), and its value is distributed in [−π,π]. For *N* radar SLC images in a time series, differential interference processing is performed on each target point at different times:

(1) $$\Delta \varphi_{n}=\mathrm{angle}\left(s_{i}e^{j\varphi_{i}}{\left(s_{j}e^{j\varphi_{j}}\right)}^{*}\right)=\mathrm{angle}\left(s_{i}s_{j}e^{j(\varphi_{i}-\varphi_{j})}\right)$$

where $\Delta \varphi_{n}$ is the interference phase of the *n* point, $P_{i}=s_{i}e^{j\varphi_{i}}$ is the *i* radar echo image, $P_{j}=s_{j}e^{j\varphi_{j}}$ is the *j* radar echo image, and the symbol ∗ is conjugate. The interference phase of the *n* target point in the differential interferogram can be modeled as

(2) $$\Delta \varphi =\Delta \varphi_{\mathrm{dis}}+\Delta \varphi_{\mathrm{atm}}+\Delta \varphi_{\mathrm{noise}}+2k\pi$$

where $\Delta \varphi_{\mathrm{dis}}$ is the LOS variable, $\Delta \varphi_{\mathrm{atm}}$ is the atmospheric delay phase introduced by the change in atmospheric refractive index at different times, $\Delta \varphi_{\mathrm{noise}}$ is the phase of incoherent noise, which can be filtered by using a low-pass phase filter. 2kπ is the fuzzy phase, and *k* is an integer, which represents the ambiguity [24].

However, it should be noted that in this experiment, we assume that the error phase, such as the atmospheric phase, has little effect, and our deformation monitoring target is a strong scatterer, which is less affected by the error. Therefore, the method proposed in this paper does not consider the influence of atmospheric phase error and random noise error and directly solves the deformation through the interference phase obtained by GB-SAR [25].

GB-SAR deformation monitoring technology uses differential interferometry. This technique fuses and analyzes two SAR complex images obtained by radar in the same target area. By calculating the target phase information obtained by the radar at different times, the millimeter-level precision deformation information of the target is obtained. Each pixel in the image obtained by GB-SAR is a complex number, and its amplitude is usually used to interpret the imaging scene and study the scattering characteristics. When GB-SAR is used for deformation measurements, the radar position is fixed, and the spatial baseline between different images is zero. Differential interferometry can be achieved by using complex conjugate multiplication on the corresponding pixels of two images. GB-SAR is mainly used to monitor the same area on parallel orbits and obtain two (or more) SLC images to form interference [26].

**Figure 1:**
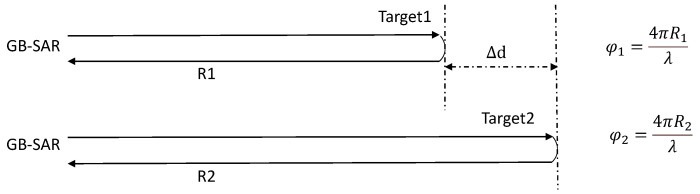
Principle of differential interferometry.

Figure 1 depicts the schematic diagram of GB-SAR differential interference deformation monitoring. Target 1 denotes the initial position of the target, whereas Target 2 corresponds to its position after displacement. The radar performs measurements on these targets both before and after deformation, capturing their respective phase information. Subsequently, the phase difference between the two is determined:

(3) $$\Delta \varphi =\varphi_{2}-\varphi_{1}=\frac{4\pi \left(R_{2}-R_{1}\right)}{\lambda }$$

By leveraging the correlation between phase difference and distance, we can extract the deformation information of the target projected in the LOS direction of the GB-SAR:

(4) $$\Delta d=R_{2}-R_{1}=\frac{\Delta \varphi \lambda }{4\pi }$$

where Δφ is the phase difference, λ is the wavelength of the radar, and Δd is the deformation information of the target projected in the LOS direction.

## 3. Methodology

### 3.1. Method Flowchart

**Figure 2:**
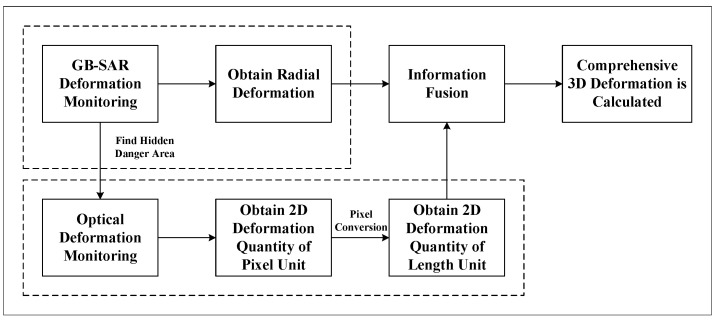
The process of three-dimensional deformation monitoring.

Since optical deformation monitoring can provide information within the plane perpendicular to the line of sight, we aim to utilize optical images to supplement the three-dimensional deformation information that cannot be obtained through radar monitoring. The workflow of the proposed method is shown in Figure 2. The main steps include radial deformation monitoring based on GB-SAR, optical deformation monitoring, pixel deformation conversion to deformation under the basic length unit, data information fusion, and three-dimensional deformation calculation.

### 3.2. Optical Deformation Monitoring

The deformation monitoring technology based on optical images is a method that can be used to monitor the deformation of objects by using optical measurement principles and imaging technology [21]. This method obtains two or more optical images before and after the deformation of the target and then analyzes the information in the image to obtain the deformation information. Deformation monitoring technology based on optical images is widely used in materials science, physics, medicine, and other fields. Optical images are used to measure the expansion, bending, and torsion of materials. The digital image correlation method is one of the most commonly used optical deformation monitoring methods. This method uses two digital images before and after the deformation of the specimen to obtain the deformation information of the region of interest through correlation calculation [27]. The basic principle is that the region of interest in the image before deformation is meshed, and the divided sub-regions are regarded as rigid motion. Then, for the selected sub-region, the correlation calculation is carried out according to the pre-defined correlation function by using a certain method, and the region with the maximum cross-correlation coefficient with the sub-region is found in the deformed image, that is, the position of the sub-region after deformation, and then the displacement of the sub-region is obtained. This method has the advantages of a strong anti-interference ability and high measurement accuracy [28].

The goal of correlation calculation is to assess the similarity between the template and the target image, underscoring the significance of choosing an appropriate correlation function in the digital image correlation method. Common correlation functions include direct correlation, normalized correlation, and covariance correlation functions. The expression for the correlation function is defined as follows:

(5) I(X,Y)=H(X)+H(Y)−H(X,Y)

where H(X) represents the information entropy of picture *X*; the expression used for its calculation is

(6) $$p_{i}=\frac{s_{i}}{\sum_{i=1}^{N-1}s_{i}}$$

(7) $$H\left(X\right)=-\sum_{i=0}^{N-1}p_{i}\log p_{i}$$

where $s_{i}$ represents the total number of pixels in image *X* with a grayscale value of *i*, *N* represents the total number of grayscale levels in image *X*, and $p_{i}$ represents the probability of grayscale *i* appearing. H(X,Y) represents the joint entropy of the two images, *X* and *Y*:

(8) $$H\left(X,Y\right)=-\sum_{x,y}p_{XY}\left(x,y\right)\log p_{XY}\left(x,y\right)$$

and I(X,Y) represents the mutual information of two pictures: *X* and *Y*. Given the suboptimal anti-noise performance of the direct correlation function, the commonly employed alternatives are the normalized correlation function and the covariance correlation function:

(9) $$EI\left(X,Y\right)=\frac{H(X)+H(Y)}{H(X,Y)}$$

(10) $$C\left(X,Y\right)=\frac{2I(X,Y)}{H(X)+H(Y)}$$

Utilizing the correlation function calculation to identify the position with the maximum correlation coefficient allowed us to determine the initial location of the target in the optical image prior to any target displacement. This enables the calculation of the pixel-level deformation of the target in the two-dimensional optical plane.

### 3.3. Pixel Conversion Based on Fitting Regression

#### 3.3.1. The Proposed Idea

The optical deformation monitoring methods represented by the digital image correlation method have many advantages. However, these optical deformation monitoring methods can only obtain pixel-level deformation, which is widely used in materials science, physics, and other fields. In these fields, it is usually only necessary to qualitatively analyze the deformation of the target or to use the size of the target as a priori known information so as to roughly convert the pixel deformation into the actual deformation. Whether it is a qualitative analysis using pixel deformation quantity or rough conversion using prior information, these methods cannot be applied to physical target deformation monitoring in actual scenes. Therefore, how to apply optical deformation monitoring to the actual deformation monitoring field and convert the pixel deformation measured at any position into actual deformation becomes a difficult problem.

**Figure 3:**
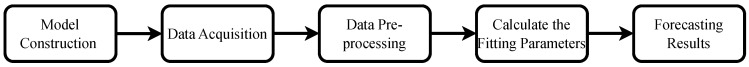
The basic process of pixel conversion based on fitting regression.

In this paper, a method based on regression fitting is proposed to convert the pixel deformation quantity obtained by optical deformation monitoring into the deformation quantity under the basic length unit. By fitting the optical sensor’s optical deformation and monitoring the data of a series of standard samples with a known detection distance, the ratio of pixel deformation at different positions in the fitting range to the deformation under the basic length unit is predicted and obtained. The basic process of the method is shown in Figure 3:

Among them, data acquisition is mainly used to collect the diagonal pixel values of standard samples of the same size at different positions. Data preprocessing mainly includes steps such as image interpolation and edge detection. The prediction result is to obtain the predicted diagonal pixel values of standard samples at different positions based on the fitting model so as to calculate the pixel conversion ratio and finally convert the pixel-level optical deformation into the deformation value under the basic length unit.

#### 3.3.2. Construction Model

The nonlinear transformation observed in camera imaging primarily stems from the multiple refractions occurring within the lens system. Among the components of the imaging system, the lens plays a pivotal role, and the optical transformation within a single lens involves phase transformation:

(11) $$t\left(x,y\right)=\exp\left(-jk\frac{x^{2}+y^{2}}{2f}\right)$$

It can be seen that the influence of a single lens on optics is an exponential function relationship. Therefore, when nonlinearly fitting camera imaging, an exponential function model should be established:

(12) f(P)=a∗exp(b∗R)

where *R* is the object distance, and *P* is the diagonal pixel of the standard sample when the detection distance is *R*. Since a single lens usually contains multiple sets of lenses in actual imaging, in order to obtain better fitting prediction results, the constructed nonlinear fitting model should contain at least two sets of exponential functions:

(13) $$f\left(P\right)=ae^{\left(b*R\right)}+ce^{\left(d*R\right)}$$

After obtaining the diagonal pixels of the standard sample at the detection distance $R_{i}$, the pixel conversion ratio *r* can be calculated here. The expression of the pixel conversion ratio is defined as

(14) $$r=\frac{D}{P}\left(R=R_{i}\right)$$

where both *D* and *P* denote the diagonal dimensions of the plane containing a set of identical standard samples at a detection distance, $R_{i}$, where *D* is measured in millimeters (mm), and *P* is measured in pixels. For the purposes of this study, the standard samples are a set of optical calibration boards with dimensions of 80 mm by 80 mm. Consequently, the diagonal size (*D*) of this standard sample can be calculated using the formula $D=\sqrt{80^{2}+80^{2}}$ mm.

For the acquisition of detection distance, the ranging function of GB-SAR can be used. In the actual deformation monitoring scene, GB-SAR can directly read the detection distance of the corresponding position after monitoring the deformation point. When the optical sensor is placed in the radar center of GB-SAR, the detection distance obtained by GB-SAR is $R_{i}$.

Through the nonlinear fitting model, the pixel *P* of the standard sample at any $R=R_{i}$ position can be obtained by using Formula (13), and then the pixel conversion ratio can be obtained by using Formula (14) so as to realize the function of converting the pixel deformation obtained in Section 3.3.1 based on an optical image under deformation and the basic length.

### 3.4. Image Fusion

**Figure 4:**
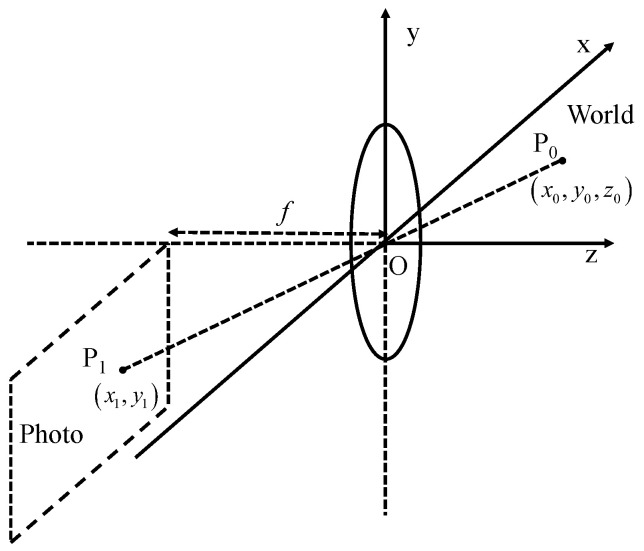
Schematic diagram of optical imaging co-ordinate transformation.

Compared with spaceborne SAR imaging, GB-SAR imaging has the problem of a large detection range span, so there is serious spatial variation in radar signal processing, and there is obvious geometric distortion in its imaging [29]. Consequently, the conventional approach of fusing spaceborne SAR and optical images is not applicable to GB-SAR and optical image fusion. This paper presents a method for combining GB-SAR and optical images through the utilization of co-ordinate system transformation, achieved by employing transformation matrices and control points [30]. Firstly, there is a target point, $P_{0}$, in the actual scene, and its co-ordinates are $\left(x_{0},y_{0},z_{0}\right)$. In the optical image, the point corresponding to $P_{0}$ is $P_{1}$, and its co-ordinates are $\left(x_{1},y_{1}\right)$, as shown in Figure 4.

According to the lens imaging principle of optical cameras, it can be seen that

(15) $$-\frac{x_{1}}{f}=\frac{x_{0}}{z_{0}}$$

(16) $$-\frac{y_{1}}{f}=\frac{y_{0}}{z_{0}}$$

where *f* is the focal length of the camera; the transformation relationship between the actual scene and the optical image can be obtained:

(17) $$\left[\begin{array}{c} x_{1}\\ y_{1}\\ 1 \end{array}\right]=-\frac{f}{z_{0}}\left[\begin{array}{ccc} 1 & & \\ & 1 & \\ & & -\frac{z_{0}}{f} \end{array}\right]\left[\begin{array}{c} x_{0}\\ y_{0}\\ 1 \end{array}\right]=-kA\left[\begin{array}{c} x_{0}\\ y_{0}\\ 1 \end{array}\right]\;$$

where matrix *A* can be proved to be an invertible matrix. The corresponding point of the target point $P_{0}$ on the GB-SAR image is $P_{2}$, and the co-ordinate under the rectangular co-ordinate system is $\left(x_{2},y_{2}\right)$. When the azimuth resolution of the GB-SAR is $\rho_{a}$, and the range resolution is $\rho_{r}$, the transformation relationship between the actual scene and the optical image can be obtained:

(18) $$\left[\begin{array}{c} x_{2}\\ y_{2}\\ 1 \end{array}\right]=\left[\begin{array}{ccc} \frac{1}{\rho_{a}} & & \\ & \frac{1}{\rho_{r}} & \\ & & 1 \end{array}\right]\left[\begin{array}{c} x_{0}\\ y_{0}\\ 1 \end{array}\right]=B\left[\begin{array}{c} x_{0}\\ y_{0}\\ 1 \end{array}\right]\;$$

It can also be proved that matrix *B* is invertible; thus

(19) $$\left[\begin{array}{c} x_{1}\\ y_{1}\\ 1 \end{array}\right]=-kAB^{-1}\left[\begin{array}{c} x_{2}\\ y_{2}\\ 1 \end{array}\right]=C\left[\begin{array}{c} x_{2}\\ y_{2}\\ 1 \end{array}\right]\;$$

Matrix *C* is the transformation matrix, which can be calculated by control points. In practical data collection scenarios, typically four–six corner reflectors are employed as pre-defined control points. Following the acquisition of both optical and GB-SAR images, the co-ordinate information of these control points can be manually extracted from the images.

### 3.5. Construct a Three-Dimensional Solution Model

**Figure 5:**
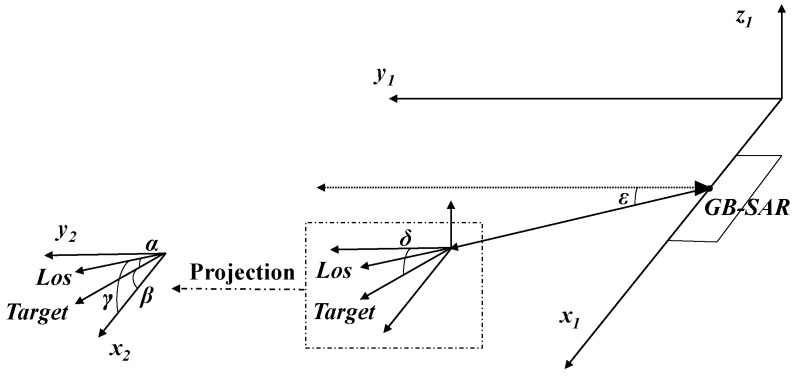
Three-dimensional deformation model.

After obtaining the radial deformation and two-dimensional plane deformation of the target, the actual three-dimensional deformation of the target can be solved by establishing a three-dimensional solution model. A basic three-dimensional deformation solution model is shown in Figure 5.

Where δ is the pitch angle of the target, and ε is the pitch angle of GB-SAR LOS, projecting the target deformation and radar LOS to the plane; in the horizontal plane, α is the angle between the radar radial direction and the target deformation direction, β is the target azimuth, and γ is the azimuth angle of GB-SAR. The three-dimensional deformation solution can be realized by using the following formula:

(20) dtsinδ=dz

(21) dtcosδcosβ=dx

(22) dtcosδcosα=dlcosε

(23) $$dt^{2}=dx^{2}+dy^{2}+dz^{2}$$

where dx, dz, and dl are the monitoring results for the X-axis, Z-axis, and radar radial direction, respectively, and dt is the real deformation of the target. Finally, the radial deformation measured by GB-SAR and the plane deformation measured by the optical camera are substituted into the model to obtain the complete deformation, dt.

## 4. Experiments and Analysis

In order to demonstrate the accuracy and efficacy of the method detailed in this paper, we carried out experiments outdoors to obtain measured data for verification. Specifically, Section 4.1 examines the pixel conversion method’s effectiveness, Section 4.2 examines the impact of image fusion, and Section 4.3 shows the obtainment of the comprehensive three-dimensional deformation quantity of the target.

### 4.1. Optical Deformation Monitoring Based on Fitting Regression Models

**Figure 6:**
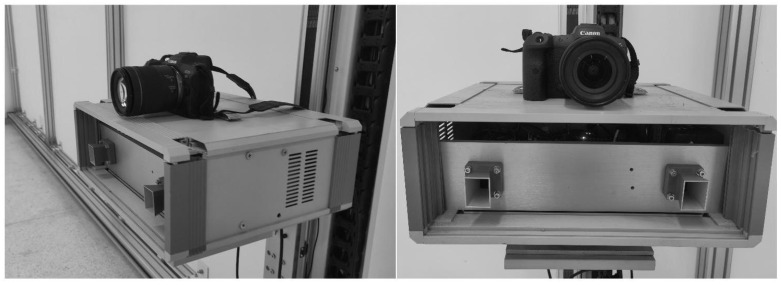
Optical GB-SAR deformation monitoring system.

Firstly, the fitting parameters of Formula (13) were solved by practical experiments. In order to obtain the detection range of the targets, the experimental system includes an X-band GB-SAR system and an optical camera, as shown in Figure 6.

The parameters of the system are shown in Table 1.

**Figure 7:**
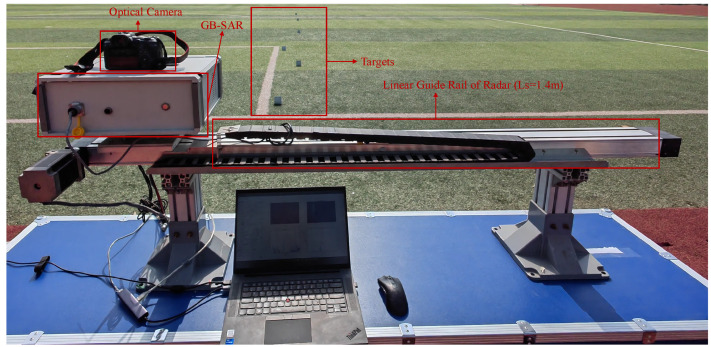
Optical deformation monitoring scene.

The lens of the optical camera is placed on the center of the radar line of sight of the GB-SAR so that the GB-SAR can obtain information such as the detection range and azimuth angle of the targets. The experimental scene is shown in Figure 7.

Identical optical calibration plates are placed in the experimental site as experimental targets. The pattern size of the optical calibration plate is 80 × 80 mm, and its diagonal size under the basic length unit is *D* = 113.12 mm. The detection distance of these optical calibration plates is collected for fitting parameters. The detection distance and diagonal pixels of the six calibration boards are obtained, as shown in Table 2.

By using the nonlinear model in 3.3.2 to fit the parameters *a* = 756.3, *b* = −0.5089, *c* = 109.3, *d* = −0.04932, the nonlinear regression model in the fitting range is obtained:

(24) $$f\left(P\right)=756.3e^{\left(-0.5089R\right)}+109.3e^{\left(-0.04932R\right)}$$

Repeat the experiment by changing the position of the calibration plate to obtain the detection distance and diagonal pixels of the six calibration plates and compare them with the fitting prediction data of Equation (24), as shown in Table 3:

The root mean square error (RMSE) and mean relative error (MRE) of the model are calculated, as shown in Table 4.

It can be seen that the nonlinear fitting model can be reused after a parameter solution. The root mean square error is 0.9301 mm, and the average relative error can be controlled within 3%. It shows that the prediction effect of the fitting model is good. In order to verify the effect of the pixel conversion method in the actual optical deformation monitoring, a corner reflector is used as the target to move horizontally by 3.0 mm in the experimental site to obtain two images before and after deformation.

**Figure 8:**
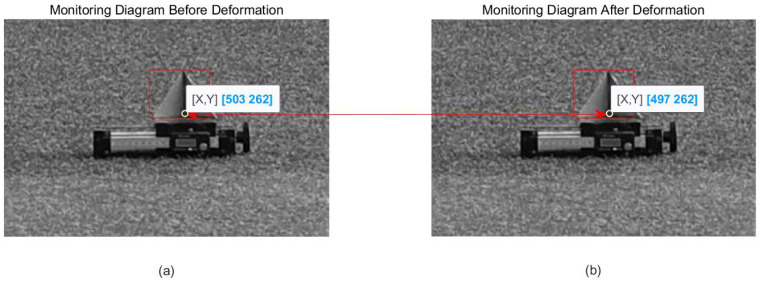
Optical deformation monitoring result: (**a**) before deformation; (**b**) after deformation.

After three interpolation runs of the image, the pixel deformation of the target obtained by optical deformation monitoring is six pixels, as shown in Figure 8.

**Figure 9:**
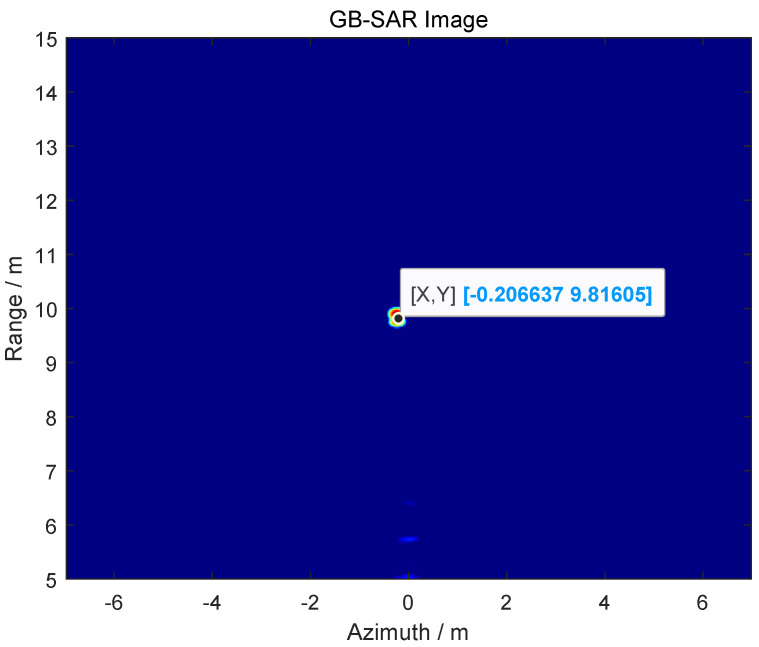
GB-SAR imaging to obtain detection range.

The position information of the target obtained by using GB-SAR is *R* = 9.82 m, as shown in Figure 9.

Based on the nonlinear fitting model of Section 3.3, the diagonal pixels of the standard samples at this position are obtained as follows:

(25) $$f\left(P\right)=756.3e^{\left(-0.5089R\right)}+109.3e^{\left(-0.04932R\right)}=72.45$$

Thus, the original pixel conversion ratio here is 113.12/72.45 = 1.56. Due to the interpolation of the optical monitoring image by a factor of three, the pixel conversion ratio in the image is *r* = 1.56/3 = 0.52. Consequently, the pixel deformation quantity obtained from optical deformation monitoring is converted into the deformation quantity under the basic length unit as 6 × 0.52 = 3.12 mm. The relative error between the result and the actual movement of the target is 4%.

In order to better test the performance of the method, we changed the deformation distance of the target, repeated the experiment, and obtained the results shown in Table 5:

It can be seen that the accuracy of optical deformation monitoring using this method can reach a sub-millimeter level within 10 m, and the relative error can be controlled within 6%. In order to better verify the applicability and effectiveness of the proposed method, the detection distance was increased, and the results obtained by repeating the above experiments are shown in Table 6:

It can be seen that the accuracy of optical deformation monitoring using this method can reach the millimeter level at a detection distance of tens of meters, and the relative error can be controlled within 7%.

The experiments show that the method proposed in this paper realizes the real-time pixel conversion function at any position in the range of several meters to dozens of meters. The method does not need to solve the parameters again after solving the model parameters once and can be reused in subsequent experiments. It solves the problem of the pixel deformation quantity is difficult to convert into deformation quantity under a basic length unit in optical deformation monitoring, and it greatly improves the applicability and practicability of the whole deformation monitoring system.

### 4.2. Image Fusion

**Figure 10:**
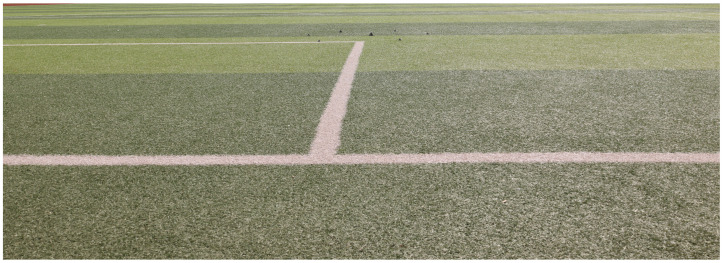
Optical image of some corner reflectors.

In order to verify the effectiveness of the proposed algorithm in Section 3.4, we carried out multi-objective image fusion verification experiments outdoors. Firstly, some corner reflector targets are randomly arranged in the experimental site, as shown in Figure 10.

**Figure 11:**
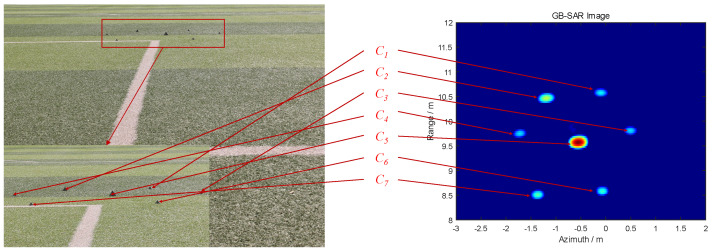
GB-SAR image and an optical image of the corner reflectors.

The GB-SAR image and an optical image of these corner reflectors are depicted in Figure 11, where $C_{n}$ represents the corresponding position of the nth corner reflector in the optical and radar image.

**Figure 12:**
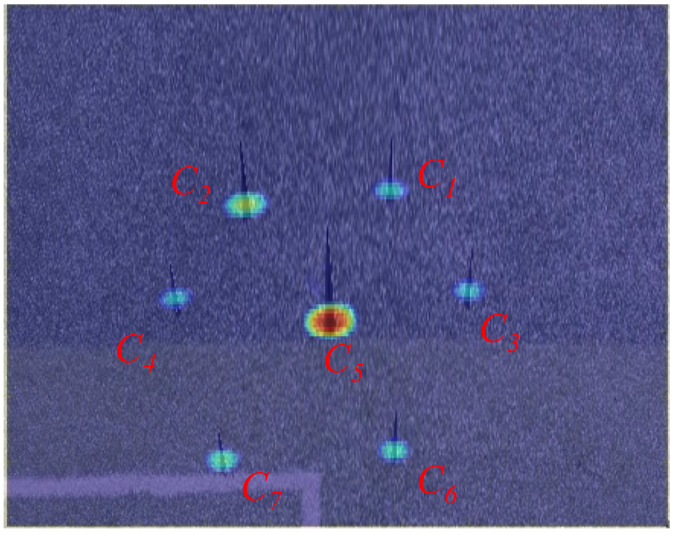
Fusion image.

Based on the algorithm in Section 3.4, the images are merged, as shown in Figure 12.

The enhanced fusion result is evident, displaying improved alignment between the targets in both the GB-SAR and optical images. No significant dislocation is observed, resulting in a more natural and clear visualization effect. The fusion of GB-SAR and optical images allows for the identification of potential hazards through radar, followed by the selection of any corresponding areas in the optical image for optical deformation monitoring. Subsequently, the fusion data from GB-SAR radial deformation monitoring and optical image two-dimensional deformation monitoring are utilized to calculate the comprehensive three-dimensional deformation data of the target.

### 4.3. Obtaining Three-Dimensional Deformation Quantity

**Figure 13:**
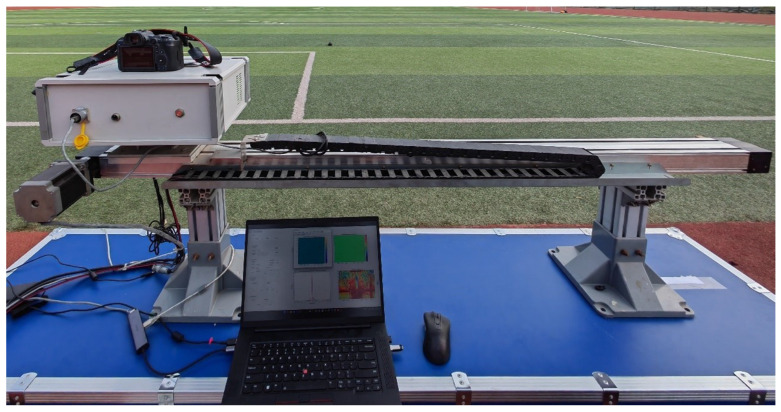
Three-dimensional deformation monitoring experimental scene.

In order to verify the feasibility of the method proposed in this paper, we carried out data acquisition experiments in actual outdoor scenes to obtain the three-dimensional deformation monitoring amount of the target; the experimental scene is shown in Figure 13.

**Figure 14:**
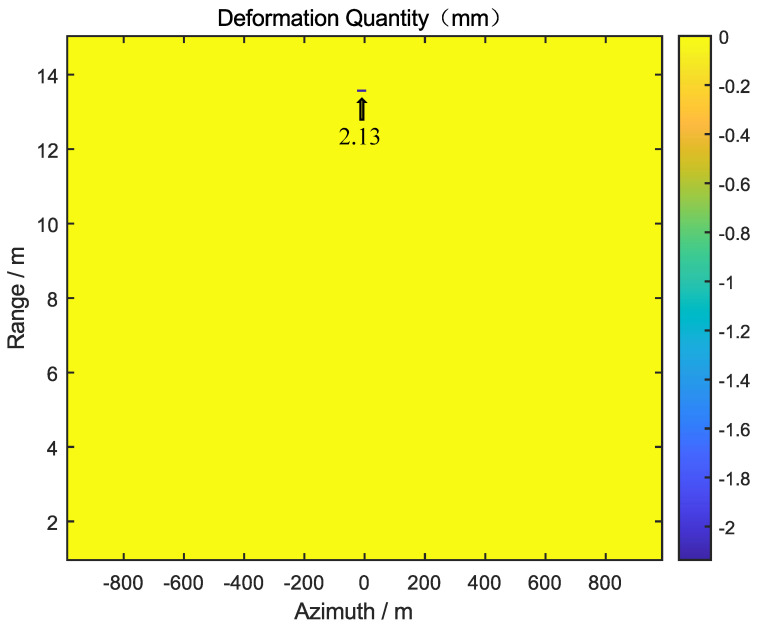
GB-SAR acquires the radial deformation of the target.

By moving the target along the X, Y, and Z axes by 2.0 mm, 2.0 mm, and 2.5 mm, respectively, the actual three-dimensional deformation of the target is 3.77 mm. First, the GB-SAR was used to monitor the radial deformation of the target, and the radial deformation, dl, of the target is 2.13 mm, as shown in Figure 14.

**Figure 15:**
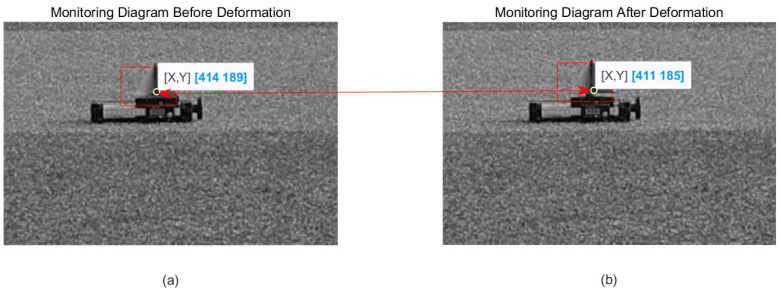
Optical deformation monitoring result: (**a**) before deformation; (**b**) after deformation.

After that, the optical camera is used to obtain the optical two-dimensional deformation monitoring of the target, and the two-dimensional deformation, dx = 3 pixel and dz = 4 pixel, of the target in pixel units is obtained, as shown in Figure 15.

**Figure 16:**
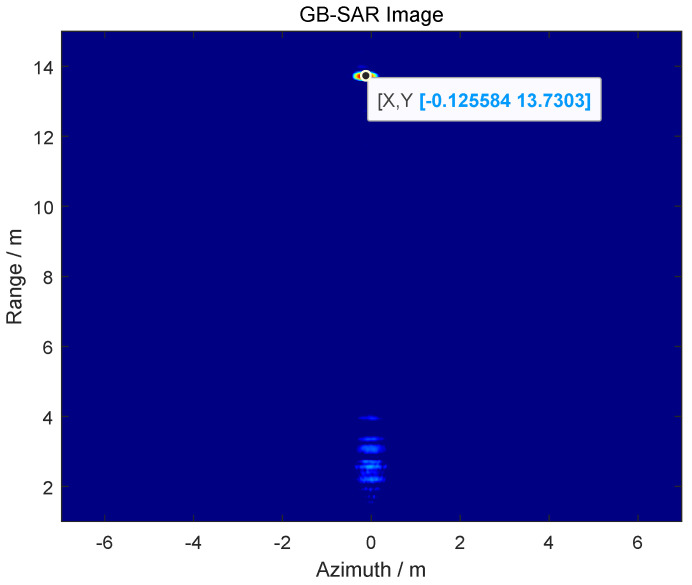
GB-SAR image of the target.

Using the ranging function of GB-SAR to obtain the detection range of the target imaging results in *R* = 13.73m, as shown in Figure 16.

Based on the pixel conversion algorithm in Section 3.3, the diagonal pixels of the calibration board at this detection distance are *P* = 56.23 pixels. Therefore, the pixel conversion ratio is *r* = 2.01. Since the actual image of optical monitoring is interpolated three times, the pixel conversion ratio in the image is *r* = 2.01 / 3 = 0.67, and then the optical deformation monitoring result—dx = 2.01 mm, dz = 2.68 mm—under the basic length unit is obtained. By substituting the optical deformation monitoring data and GB-SAR monitoring data into the three-dimensional solution model shown in Figure 5, the final comprehensive three-dimensional deformation monitoring result is 3.91 mm, and the relative error is 3.71%.

**Figure 17:**
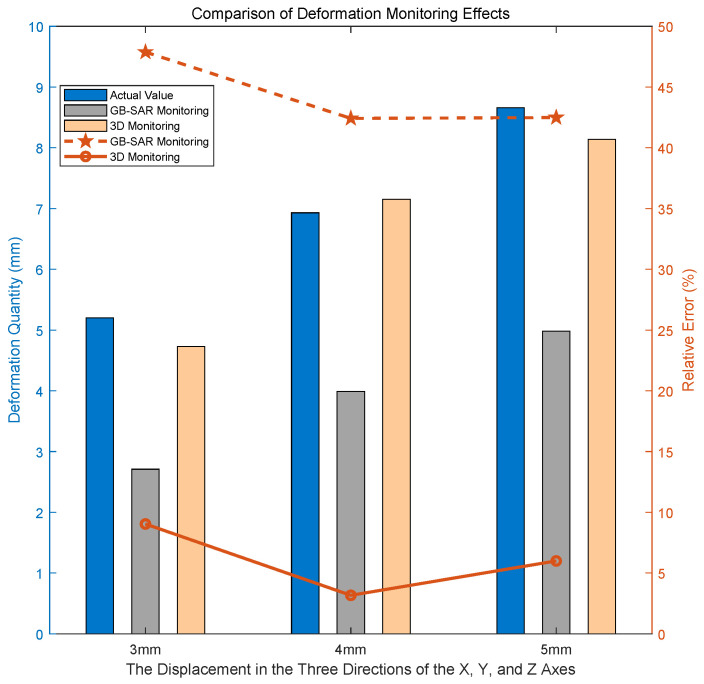
Comparison of monitoring effects at *R* = 13.73 m.

In order to better show the effect of the experiment, the target was moved the same distance along the X-axis, Y-axis, and Z-axis by 3 mm, 4 mm, and 5 mm, respectively. The comparative experimental results are illustrated in Figure 17. The term “GB-SAR Monitoring” denotes the outcomes obtained through the exclusive use of GB-SAR for deformation monitoring, and “3D Monitoring” signifies the results obtained by applying the method proposed in this paper to deformation monitoring.

When observing the results, it becomes evident that when there is a significant angle between the actual deformation direction of the target and the radial direction of the radar, the radial deformation monitored by the radar is notably underestimated compared to the real deformation. However, through the supplementation of optical image information, the three-dimensional monitoring of deformation aligns more closely with the actual deformation. This approach proves effective in preventing missed alarms resulting from substantial angles between the deformation direction and the radial direction of the radar.

**Figure 18:**
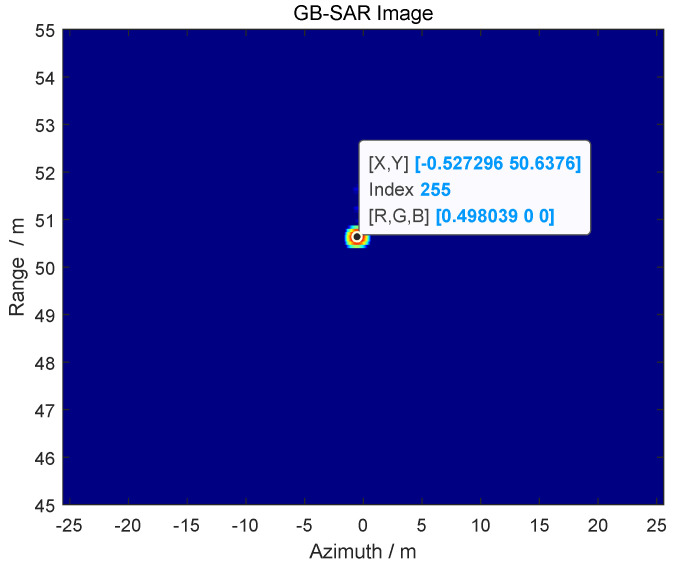
GB-SAR image of the target.

In order to better verify the applicability and effectiveness of the proposed method, the detection distance of the target was changed. The radar imaging is shown in Figure 18, and the detection distance is *R* = 50.64 m.

**Figure 19:**
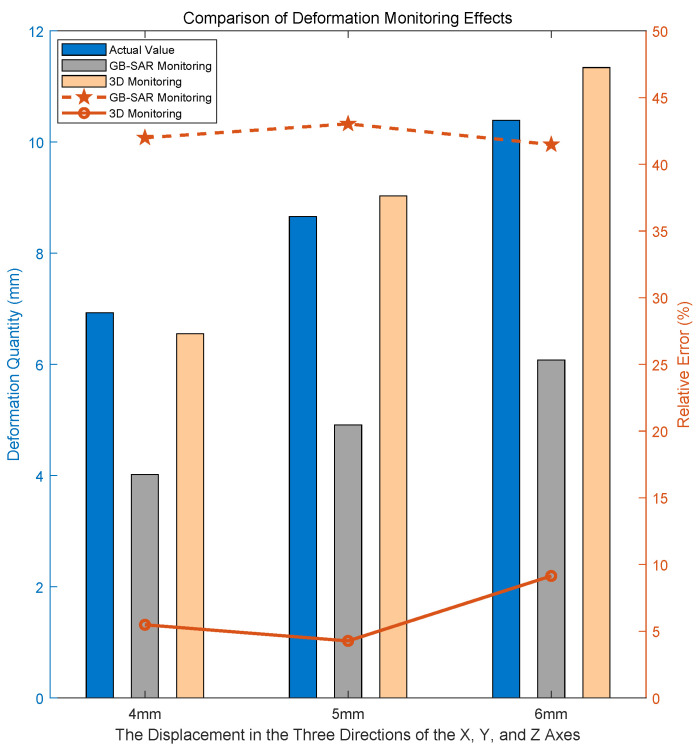
Comparison of monitoring effects at *R* = 50.64 m.

The outcomes of the repeated experiments showcasing three-dimensional deformation monitoring are illustrated in Figure 19.

The mean relative error (MRE) and root mean square error (RMSE) of the experimental results of the proposed method under different detection distances were calculated, as shown in Table 7.

Through the conducted experiments, it can be proved that the three-dimensional deformation monitoring method proposed in this paper can effectively carry out comprehensive three-dimensional deformation monitoring for targets within a monitoring range of tens of meters. Among them, sub-millimeter-level monitoring accuracy can be achieved at a detection distance of 10 meters, and millimeter-level monitoring accuracy can be achieved at a detection distance of tens of meters. The average relative error of monitoring can be controlled within 10%, and the root mean square error can be controlled within 0.7 mm, indicating that the monitoring effect of this method is good and the comprehensive three-dimensional deformation of the monitoring target can be effectively applied.

## 5. Discussions

### 5.1. Comparison of Conventional Methods and the Proposed Method

Predominantly, the research in three-dimensional deformation monitoring is centered on spaceborne and airborne SAR, with limited methodologies tailored for ground targets. Current research into the three-dimensional deformation monitoring of ground targets predominantly focuses on two main approaches. The primary research directions include multi-station three-dimensional deformation monitoring and the use of prior terrain information to predict deformations in various directions.

In the context of multi-station three-dimensional deformation monitoring, the predominant method entails employing multiple GB-SARs for multi-view interferometry measurements. This technique typically necessitates a minimum of two GB-SARs, and for enhanced accuracy, three GB-SARs are often concurrently deployed. However, the current accuracy level is confined to the centimeter range, accompanied by substantial costs and deployment challenges, significantly impeding the widespread adoption of GB-SARs.

In contrast, the three-dimensional deformation method proposed in this article requires only single-station monitoring. This significantly diminishes the cost and deployment complexities associated with three-dimensional deformation monitoring when compared to the aforementioned method.

Additionally, in contrast to predicting deformation information using prior terrain information, which relies on prior knowledge of the terrain and time series analysis combined with geographic reference models, the proposed method in this article operates without the need for geographic reference models or similar data. This increased flexibility enhances its adaptability across diverse scenarios, effectively expanding the application range of GB-SAR.

### 5.2. Conflict between Accuracy and Monitoring Distance

The accuracy of the three-dimensional deformation monitoring method proposed in this article decreases with the increase in monitoring distance, as demonstrated by a series of experiments in Section 4.3. The main reason for this problem is limited by the current constraints of optical imaging equipment. In theory, the current method can achieve millimeter-level accuracy in three-dimensional deformation monitoring at tens of meters and can control the relative error within 10%, as illustrated in Figure 17 and Figure 19 and Table 7. However, as the detection distance increases to the 100-meter range, the accuracy of the three-dimensional deformation monitoring method will decrease to the centimeter level. With the rapid development of optical devices, long-distance three-dimensional deformation monitoring will gradually be carried out. At that time, the atmospheric phase correction work mentioned in Formula (2) will also come into the picture, further enhancing the applicability of the method in this article.

## 6. Conclusions

This paper introduces a new method to obtain the three-dimensional deformation of a target based on ground-based synthetic aperture radar deformation monitoring and optical deformation monitoring. This method only needs single-station monitoring, which avoids the high cost and difficult deployment of dual-station monitoring. It can obtain a comprehensive three-dimensional deformation of the ground-based target. Compared with the traditional use of GB-SAR alone for deformation monitoring, it can effectively reduce the monitoring error and avoid potential safety hazards such as missed alarms. Through outdoor physical acquisition experiments, the proposed method demonstrates sub-millimeter monitoring accuracy within a 10-meter range and millimeter-level accuracy at a range of tens of meters. The average relative error can be controlled within 10%, and the root mean square error can be controlled within 0.7 mm. It can be effectively applied to the three-dimensional deformation monitoring of foundation targets field and provides a new idea for natural disaster warnings and building deformation monitoring.

## Figures and Tables

**Table 1:** Index parameters of the system.

Optical-GB-SAR Deformation Monitoring System	
Bandwidth	4 GHz
Center Frequency	10 GHz
Length of Synthetic Aperture	1.4 m
Range Resolution	0.0375 m
Dimensions of Photowriting Sensor	36 × 24 mm
Active Picture Elements	4500 w
Focus Range	−3∼+19 EV

**Table 2:** Obtaining fitting data.

	Target 1	Target 2	Target 3	Target 4	Target 5	Target 6
Detection Range (m)	1.90	3.90	6.70	12.29	27.57	40.99
Diagonal Pixels	387.67	191.33	108.45	61.21	26.60	15.70

**Table 3:** Comparison of fitting prediction effect.

	Target 1	Target 2	Target 3	Target 4	Target 5	Target 6
Detection Range (m)	14.50	18.05	21.90	24.00	41.00	45.20
Diagonal Pixels	52.85	43.40	37.32	33.27	15.70	11.93
Predicted Pixels	53.93	44.95	37.12	33.47	14.47	11.76

**Table 4:** Comparison of fitting prediction effect.

RMSE (mm)	0.9301
MRE	2.67%

**Table 5:** Optical deformation monitoring results with a detection distance of 9.82 m.

Deformation Distance (mm)	Monitoring Distance (mm)	Relative Error
3.0	3.1	3.3%
3.5	3.6	2.9%
4.0	4.2	5.0%

**Table 6:** Optical deformation monitoring results with a detection distance of 55.62 m.

Deformation Distance (mm)	Monitoring Distance (mm)	Relative Error
3.0	3.2	6.6%
5.0	4.8	4.0%
7.0	6.4	5.7%

**Table 7:** The monitoring effect under different detection distances.

Detection Distance (m)	MRE	RMSE (mm)
13.76	6.07%	0.5932
50.64	6.30%	0.6282

## Data Availability

Data are contained within the article.

## References

[1] Wang Y., Hong W., Zhang Y., Lin Y., Li Y., Bai Z., Zhang Q., Lv S., Liu H., Song Y. (2020). Ground-based differential interferometry SAR: A review. IEEE Geosci. Remote Sens. Mag..

[2] Zhang Z., Suo Z., Tian F., Qi L., Tao H., Li Z. (2022). A Novel GB-SAR System Based on TD-MIMO for High-Precision Bridge Vibration Monitoring. Remote Sens..

[3] Ruiz J.J., Lemmetyinen J., Lahtinen J., Uusitalo J., Häkkilä T., Kontu A., Pulliainen J., Praks J. Investigation of cryosphere processes in the boreal forest zone using ground-based SAR. Proceedings of the 2022 52nd European Microwave Conference (EuMC).

[4] Mo Y., Lai T., Wang Q., Huang H. (2023). Modeling and compensation for repositioning error in discontinuous GBSAR monitoring. IEEE Geosci. Remote Sens. Lett..

[5] Nie Q., Sun B., Li Z. Resolution analysis of sector scan GB-SAR for wide landslides monitoring. Proceedings of the 2015 IEEE International Geoscience and Remote Sensing Symposium (IGARSS).

[6] Deng Y., Tian W., Xiao T., Hu C., Yang H. (2021). High-Quality Pixel Selection Applied for Natural Scenes in GB-SAR Interferometry. Remote Sens..

[7] Chan Y., Koo V., Hii W.H., Lim C. (2021). A Ground-Based Interferometric Synthetic Aperture Radar Design and Experimental Study for Surface Deformation Monitoring. IEEE Aerosp. Electron. Syst. Mag..

[8] Leva D., Nico G., Tarchi D., Fortuny-Guasch J., Sieber A.J. (2003). Temporal analysis of a landslide by means of a ground-based SAR interferometer. IEEE Trans. Geosci. Remote Sensing.

[9] Zhan D., Yu L., Xiao J., Chen T. (2015). Multi-camera and structured-light vision system (MSVS) for dynamic high-accuracy 3D measurements of railway tunnels. Sensors.

[10] Zhu Y., Xu B., Li Z., Li J., Hou J., Mao W. (2023). Joint Estimation of Ground Displacement and Atmospheric Model Parameters in Ground-Based Radar. Remote Sens..

[11] Sun H., Zhang Q., Zhao C., Yang C., Sun Q., Chen W. (2017). Monitoring land subsidence in the southern part of the lower Liaohe plain, China with a multi-track PS-InSAR technique. Remote Sens. Environ..

[12] Xue F., Lv X., Dou F., Yun Y. (2020). A review of time-series interferometric SAR techniques: A tutorial for surface deformation analysis. IEEE Geosci. Remote Sens. Mag..

[13] Wang P., Xing C., Pan X., Zhou X., Shi B. (2021). Microdeformation monitoring by permanent scatterer GB-SAR interferometry based on image subset series with short temporal baselines: The Geheyan Dam case study. Measurement.

[14] Takahashi K., Matsumoto M., Sato M. (2013). Continuous observation of natural-disaster-affected areas using ground-based SAR interferometry. IEEE J. Sel. Top. Appl. Earth Observ. Remote Sens..

[15] Kang X., Zhu J., Geng L.Y. (2017). Application of InSAR Technique to Monitor Time-series Displacements of Transmission Towers Located in Mining Area. Electr. Power Surv. Des..

[16] Mo Y., Lai T., Wang Q., Huang H. Study on Repositioning Error Model in GBSAR Discontinuous Observation for Building Deformation Monitoring. Proceedings of the IGARSS 2023–2023 IEEE International Geoscience and Remote Sensing Symposium.

[17] Meinan Z., Yixuan L., Kazhong D., Chenliang Z., Jun F. (2017). Monitoring and prediction of railway deformation based on DinSAR and probability integral method. Bull. Surv. Mapp..

[18] Borah S.B., Chatterjee R.S., Thapa S. (2017). Detection of underground mining induced land subsidence using Differential Interferometric SAR (D-InSAR) in Jharia coalfields. Adbu J. Eng. Technol..

[19] Lin Y., Liu Y., Wang Y., Ye S., Zhang Y., Li Y., Li W., Qu H., Hong W. (2020). Frequency domain panoramic imaging algorithm for ground-based ArcSAR. Sensors.

[20] Deng Y., Hu C., Tian W., Zhao Z. (2020). 3-D deformation measurement based on three GB-MIMO radar systems: Experimental verification and accuracy analysis. IEEE Geosci. Remote Sens. Lett..

[21] Jiang Z., Kemao Q., Miao H., Yang J., Tang L. (2015). Path-independent digital image correlation with high accuracy, speed and robustness. Opt. Lasers Eng..

[22] Yang D., Buckley S.M. (2011). Estimating high-resolution atmospheric phase screens from radar interferometry data. IEEE Trans. Geosci. Remote. Sens..

[23] Osmanoğlu B., Sunar F., Wdowinski S., Cabral-Cano E. (2016). Time series analysis of InSAR data: Methods and trends. ISPRS-J. Photogramm. Remote Sens..

[24] Baffelli S., Frey O., Hajnsek I. (2020). Geostatistical analysis and mitigation of the atmospheric phase screens in Ku-band terrestrial radar interferometric observations of an Alpine glacier. IEEE Trans. Geosci. Remote Sensing.

[25] Nico G., Cifarelli G., Miccoli G., Soccodato F., Feng W., Sato M., Miliziano S., Marini M. (2018). Measurement of pier deformation patterns by ground-based SAR interferometry: Application to a bollard pull trial. IEEE J. Ocean. Eng..

[26] Cheng Y., Huang H., Lai T., Ou P. An Image Fusion-Based Multi-Target Discrimination Method in Minimum Resolution Cell for Ground-Based Synthetic Aperture Radar. Proceedings of the 2023 4th International Seminar on Artificial Intelligence, Networking and Information Technology (AINIT).

[27] Eremin A., Lyubutin P., Panin S., Sunder R. (2022). Application of digital image correlation and Williams series approximation to characterize mode I stress intensity factor. Acta Mech..

[28] Rokoš O., Peerlings R., Hoefnagels J., Geers M. (2023). Integrated digital image correlation for micro-mechanical parameter identification in multiscale experiments. Int. J. Solids Struct..

[29] Enomoto K., Sakurada K., Wang W., Kawaguchi N., Matsuoka M., Nakamura R. Image translation between SAR and optical imagery with generative adversarial nets. Proceedings of the IGARSS 2018–2018 IEEE International Geoscience and Remote Sensing Symposium.

[30] Hughes L.H., Marcos D., Lobry S., Tuia D., Schmitt M. (2020). A deep learning framework for matching of SAR and optical imagery. ISPRS-J. Photogramm. Remote Sens..

